# Pterostilbene in the treatment of inflammatory and oncological diseases

**DOI:** 10.3389/fphar.2023.1323377

**Published:** 2024-01-08

**Authors:** Peijun Liu, Weihua Tang, Kali Xiang, Guangcai Li

**Affiliations:** ^1^ Department of Respiratory and Critical Care Medicine, The Central Hospital of Enshi Tujia and Miao Autonomous Prefecture, Enshi, China; ^2^ Department of Radiology, The Central Hospital of Enshi Tujia and Miao Autonomous Prefecture, Enshi, China

**Keywords:** pterostilbene, anti-inflammatory, antioxidant, antitumour, oral bioavailability

## Abstract

Pterostilbene (PTS), a naturally occurring analog of resveratrol (RSV), has garnered significant attention due to its potential therapeutic effects in treating inflammatory and oncological diseases. This comprehensive review elucidates the pharmacological properties, mechanisms of action, and therapeutic potential of PTS. Various studies indicate that PTS exhibits anti-inflammatory, antioxidant, and antitumour properties, potentially making it a promising candidate for clinical applications. Its influence on regulatory pathways like NF-κB and PI3K/Akt underscores its diverse strategies in addressing diseases. Additionally, PTS showcases a favorable pharmacokinetic profile with better oral bioavailability compared to other stilbenoids, thus enhancing its therapeutic potential. Given these findings, there is an increased interest in incorporating PTS into treatment regimens for inflammatory and cancer-related conditions. However, more extensive clinical trials are imperative to establish its safety and efficacy in diverse patient populations.

## Introduction

Pterostilbene (PTS), identified as trans-3,5-dimethoxy-4-hydroxystilbene, is a natural substance mainly discovered in blueberries and the wood of pterocarpus marsupium (Rimando et al., 2004). Stilbenes, including resveratrol (RSV), PTS, and pinostilbene, are plant compounds known for their potential health benefits, but the low bioavailability of RSV can limit its effectiveness (Salehi et al., 2018). RSV exhibits environmental instability, particularly its sensitivity to ultraviolet radiation, oxygen, alkaline pH, and elevated temperatures, leading to diminished bioavailability and biological activity. Consequently, numerous RSV derivatives, especially methylated compounds, are under investigation for enhanced stability and efficacy (Liu et al., 2011; Mamalis and Jagdeo, 2017). Compared to other stilbene compounds, pterostilbene boasts higher bioavailability, potentially amplifying its nutritional advantages and leading to noteworthy clinical outcomes (Kapetanovic et al., 2011). Stilbenoids are naturally occurring phenolic chemicals found in various plant species, among which resveratrol is a well-known derivative. RSV belongs to the group of phytoalexins, which are antimicrobial substances produced by plants to combat infections (Akinwumi et al., 2018). In the metabolism of stilbenoid compounds, RSV undergoes methylation to produce PTS. This biotransformation adds methyl groups to the hydroxyl moieties of RSV. PTS, once formed, can be further metabolized *in vivo*. The combined action of phase II metabolic enzymes and gut microbiota leads to the demethylation of PTS, creating pinostilbene with a singly methylated hydroxyl group. Pinostilbene, PTS, and RSV all possess the foundational framework of a stilbene configuration (C6-C2-C6), highlighting a commonality in their chemical frameworks. These phytoalexins are antimicrobial substances synthesized by plants when they come under attack by pathogens, thereby playing a significant role in the plant’s defense mechanism (Ahuja et al., 2012; Jeandet et al., 2013).

**FIGURE 1:**
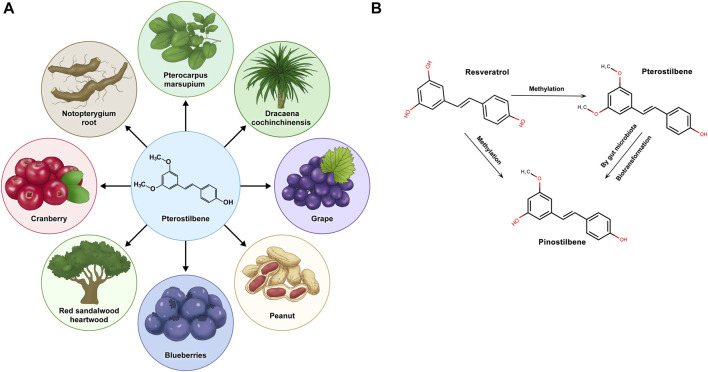
Pterostilbene is derived from natural plants and transforms within the organism. **(A)** Pterostilbene is sourced from various natural plants. **(B)** Pterostilbene, along with resveratrol and pinostilbene, undergoes specific methylation processes within the physiological environment of the organism, leading to potential metabolic changes.

PTS is a prominent nonflavonoid polyphenolic compound naturally found in various plants. Characterized by its lipophilicity, PTS appears in cis and trans isomeric structures, with the trans isomer being more dominant. Although first discovered in the heartwood of sandalwood, later research has discerned its occurrence in blueberries and grapes (Kosuru et al., 2016) (Figure 1A). While both share structural similarities, adding two methyl groups to PTS grants it unique pharmacological properties distinct from RSV (Estrela et al., 2013). A notable characteristic of PTS distinguishing it from other phytoalexins is its broad spectrum of pharmacological traits, including anti-inflammatory, antioxidant, and anticancer effects (Mccormack and Mcfadden, 2013; Akinwumi et al., 2018). Moreover, scientific studies have demonstrated that, compared to its parent compound, RSV, PTS exhibits superior bioavailability and metabolic stability, thus showing potential for further therapeutic applications (Yun et al., 2014; Peng et al., 2018). Throughout time, PTS has demonstrated advantages across multiple areas, including neuroprotection, antioxidation, and anti-inflammatory and anticancer properties, positioning it as a promising subject for continued studies in health prevention. (Hougee et al., 2005; Chen et al., 2017; Abd-Elmawla et al., 2023).

Inflammation acts as a core reaction of the immune system to harm or infections. This defensive response incorporates immune cells, blood vessels, and cellular agents to address the primary source of cellular damage, remove harmed cells and tissues, and start the process of cellular and tissue recovery (Antonelli and Kushner, 2017; Roy et al., 2022). Research indicates that PTS exhibits anti-inflammatory attributes via various pathways. Experiments with animal subjects and cellular models have highlighted the inflammation-reducing capabilities of PTS, pointing to its prospective utility in addressing inflammation-related conditions (Lim et al., 2020; Lin et al., 2020; Nagarajan et al., 2022). The use of PTS in cancer treatment is just starting, but it represents a potentially sensitizing therapy that could improve the outcome of numerous oncology treatments (Obrador et al., 2021). PTS has exhibited potential benefits in hindering and treating multiple cancer forms, such as those of the breast, prostate, colon, lung, liver, and skin (Dhar et al., 2016; Kumar et al., 2017; Ma et al., 2019). Its action pathways include controlling cell cycle dynamics, triggering programmed cell death, impeding the creation of new blood vessels, and curbing the spread of cancer cells (Estrela et al., 2013; Tzeng et al., 2021). This review aims to provide a comprehensive overview of the current knowledge and applications of PTS in inflammatory and oncological diseases.

## Pharmacokinetics

PTS, a compound sourced naturally from the diet, has garnered attention because of its expansive medicinal properties (Nagarajan et al., 2022). Compared to RSV, PTS has a more stable metabolism and enhanced pharmacological activity (Wang and Sang, 2018), owing to the presence of two methoxy groups, which are absent in RSV (Kapetanovic et al., 2011) (Figure 1B). The systemic clearance rate of PTS, reflected by its half-life, is consistent across different administration methods, indicating a swift process of absorption, distribution, metabolism, and excretion (Wang and Sang, 2018). The pharmacokinetic comparison between PTS and RSV in rats, as conducted by Kapetanovic, demonstrated that PTS’s peak plasma level was significantly higher than that of RSV, also exhibiting a notably increased oral bioavailability (Kapetanovic et al., 2011). ADME, an acronym for absorption, distribution, metabolism, and excretion, serves as a crucial framework in pharmacology to assess how drugs interact within the body. This model is particularly useful in understanding the behavior of compounds such as PTS.

## Absorption of pterostilbene

After ingestion, PTS is rapidly absorbed, contributing to its superior oral bioavailability of approximately 80%–95% (Lin et al., 2020). This rapid absorption facilitates its availability for systemic circulation. The compound’s low molecular weight and two methoxy groups enhance its lipophilic nature, aiding its penetration through the blood-brain barrier and offering potential benefits to the central nervous system (Deng et al., 2015). The ability of PTS to cross the blood-brain barrier efficiently not only broadens its therapeutic scope but also makes it a promising candidate for treating a range of neurological conditions, where effective drug delivery across this barrier is often a significant challenge.

## Distribution of pterostilbene

Following absorption, PTS exhibits a distinct distribution across various tissues in C57BL/6 mice. Within 20 min of oral intake, PTS primarily concentrates in the stomach, liver, and testes, indicating significant absorption and metabolic activity. It also appears notably in the kidneys, intestines, and lungs, suggesting potential effects on excretion, digestion, and respiratory functions. Additionally, PTS is present in the brain, spleen, skeletal muscles, and heart, highlighting its systemic reach and possible impacts on neurological, immune, muscular, and cardiovascular health. This pattern reveals PTS’s diverse interactions across various organs in a descending concentration order (Wang et al., 2022). The brain’s unique metabolic response to PTS highlights its potential in neurotherapeutic applications. This compound is selectively utilized by brain tissue, suggesting efficacy in treating neurological conditions. Its ability to cross the blood-brain barrier and engage in brain metabolism underscores its suitability for targeting brain-related disorders. This selective uptake suggests Pterostilbene’s promise in developing more focused and effective neurological treatments (Azzolini et al., 2014).

## Metabolism of pterostilbene

PTS undergoes significant first-pass metabolism in the liver, which is vital for its systemic clearance. This metabolism primarily involves phase II detoxification reactions, predominantly glucuronidation, and sulfation, transforming PTS into more water-soluble forms suitable for excretion (Kapetanovic et al., 2011; Gómez-Zorita et al., 2021; Li et al., 2023). Compared to RSV, PTS has a much lower glucuronidation efficiency in the liver, affecting its human metabolism (Dellinger et al., 2014). In comparison to RSV, its structural counterpart, PTS exhibits a considerably lower efficiency in glucuronidation within the liver. This difference in glucuronidation efficiency plays a significant role in the metabolic fate of PTS in human bodies, influencing its overall metabolism and bioavailability. The lower glucuronidation rate of PTS, as opposed to RSV, potentially allows for a longer systemic presence and a prolonged therapeutic window. This characteristic of PTS metabolism is pivotal in understanding its pharmacokinetics and pharmacodynamics, providing insights into its potential advantages over RSV in clinical applications.

## Excretion of pterostilbene

The majority of Pterostilbene’s glucuronide-conjugated metabolites are excreted within 12 h post-administration, indicating rapid renal and total serum clearance (Remsberg et al., 2008). This swift elimination reduces the chances of PTS accumulation, enhancing its suitability as a therapeutic agent. The rapid clearance is particularly beneficial for treatments requiring regular dosing, ensuring stable therapeutic levels without the risk of toxicity from accumulation. This attribute allows precise control over the drug’s pharmacokinetics, enabling adjustments in dosage or frequency to suit individual patient needs while maintaining safety and efficacy. The extent of Pterostilbene’s binding to plasma proteins can significantly impact its free concentration in the bloodstream and its subsequent distribution to tissues, affecting both its efficacy and clearance rate. Overall, Pterostilbene’s pharmacokinetic profile makes it a promising candidate for safe and effective therapies, especially in cases requiring frequent administration.

## Antioxidant activity

Oxidative stress is a condition characterized by an imbalance between the generation of reactive oxygen species (ROS) and the body’s capacity to counteract or eliminate these detrimental molecules (Schieber and Chandel, 2014). This imbalance can lead to oxidative damage to cells and tissues, triggering various health issues, including inflammation, aging, and chronic diseases. To maintain physiological balance, the human body relies on antioxidant systems to neutralize ROS, such as antioxidant enzymes and antioxidants like vitamin C and vitamin E. Major ROS include hydrogen peroxide (H_2_O_2_), superoxide anion (O_2_^−^), and hydroxyl radicals (Baskaran et al., 2021). These highly reactive molecules can be produced through endogenous metabolic processes or as a result of exposure to environmental stressors. Antioxidant therapy aims to counteract oxidative stress by either neutralizing ROS or enhancing the body’s antioxidant defense mechanisms (Rahman et al., 2020). PTS, a derivative of RSV, exhibits antioxidant activity through direct and indirect mechanisms. It acts as a ROS scavenger, neutralizing harmful free radicals and preventing cellular damage linked to chronic diseases. Additionally, PTS indirectly enhances the body’s antioxidant defenses by upregulating enzymes, providing comprehensive protection against oxidative stress. This dual action of directly combating free radicals and boosting internal defense mechanisms underscores PTS’s potential as an effective compound in antioxidant therapies and chronic disease management.

**FIGURE 2:**
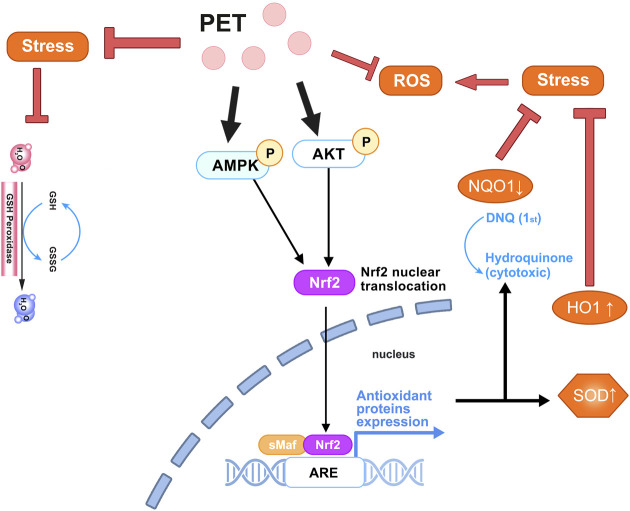
The antioxidative mechanism of pterostilbene in the physiological environment. Pterostilbene promotes the phosphorylation of AMPK and AKT, facilitates the nuclear translocation of Nrf2, and mitigates ROS, thereby alleviating oxidative stress.

PTS can reduce oxidative stress and counteract ROS like H_2_O_2_ and O_2_^−^(Mccormack and Mcfadden, 2013). PTS has been found to proficiently diminish the production of ROS in human retinal endothelial cells (HREC), particularly under high-glucose environments. PTS exerts its indirect antioxidant effects by modulating cellular pathways and enhancing the expression of crucial antioxidant enzymes. Zhou et al. showed that PTS activates the phosphorylation of AMPK and AKT, prompting the shift of Nrf2 from the cytoplasm into the nucleus. This action then heightens the expression of Nrf2-regulated genes, NQO1 and HO-1, underscoring pterostilbene’s robust antioxidant capabilities (Zhou et al., 2019). Additionally, it aids in boosting the expression of several peroxidase enzymes in diverse cellular systems, especially total glutathione, glutathione peroxidase, glutathione reductase, and superoxide dismutase (Mccormack and Mcfadden, 2013). Furthermore, as an antioxidant, PTS can neutralize harmful free radicals in the body, thereby preventing cellular damage that can lead to chronic inflammation and potential cancer (Jayakumar et al., 2021). Following the administration of PTS at a dosage of 40 mg/kg over 6 weeks, it exhibited a pronounced capability to neutralize free radicals within the system of diabetic rats, resulting in a marked decrease in oxidative stress (Amarnath Satheesh and Pari, 2006). In an *in vitro* investigation utilizing the H_2_O_2_-induced intestinal porcine enterocyte cell line (IPEC-J2), it was found that both RSV and PTS significantly ameliorated oxidative stress-induced intestinal damage. This therapeutic effect was achieved by regulating mitochondrial redox balance and functionality through the SIRT1 signaling pathway. Notably, PTS exhibited a markedly enhanced efficacy in affording this protective action in comparison to RSV (Chen et al., 2021). The antioxidant mechanism of PTS is shown in Figure 2.

## Anti-inflammatory effects of pterostilbene

Inflammation is a complex and highly orchestrated biological response that serves as a fundamental component of the body’s defense mechanism. When the body encounters harmful stimuli, including pathogens, injured cells, or irritants, a sophisticated cascade of events is triggered to protect and restore tissue integrity (Arulselvan et al., 2016). At its core, inflammation is a protective response aimed at eliminating the source of injury or infection and initiating the healing process. This multifaceted process involves the activation of immune cells, the release of signaling molecules, and the recruitment of various cellular components to the site of inflammation (Henao-Mejia et al., 2012). It is an essential part of the immune response that facilitates the healing process and repairs injured tissues. While these processes are vital for healing and defense against further damage, prolonged or widespread inflammation can be a driving force behind the emergence and advancement of many diseases (Libby et al., 2018; Sochocka et al., 2019). PTS has garnered significant attention in multiple preclinical researches for its potent anti-inflammatory properties. By targeting various stages of the inflammatory cascade, PTS effectively reduces the immediate symptoms and concurrently plays a role in averting the long-term complications typically linked with chronic inflammation. These studies suggest that the principal mechanism behind PTS’s effectiveness lies in its ability to modulate various signaling pathways intricately associated with inflammation (Kosuru et al., 2016; Wu et al., 2019).

PTS mitigates inflammation by decreasing inflammatory indicators, notably tumor necrosis factor-alpha (TNF-α), and obstructing NF-κB activation, a primary orchestrator of inflammation (Lin et al., 2016; Liu et al., 2019). By suppressing the production of TNF-α, PTS can effectively reduce the extent of the inflammatory response (Yi et al., 2022). By inhibiting the activation of NF-κB, PTS prevents this sequence of events and reduces the inflammatory response (Zhang et al., 2023). *In vitro* studies have indicated that PTS inhibits the activation of NF-κB, leading to the downregulation of proinflammatory cytokines, including TNF-α, interleukin-1beta (IL-1β), and interleukin-6 (IL-6) (Zeng et al., 2020). Utilizing its antioxidative mechanisms, PTS curbs the emergence of inflammatory markers like TNF-α, IL-1β, IL-6, MMP-2, and MMP-9 in corneal epithelial cells under hyperosmotic stress, thus shielding them from inflammation (Li et al., 2016). Overproduction of these cytokines, essential for immune reactions to pathogens and damage, often instigates excessive inflammatory responses.

**FIGURE 3:**
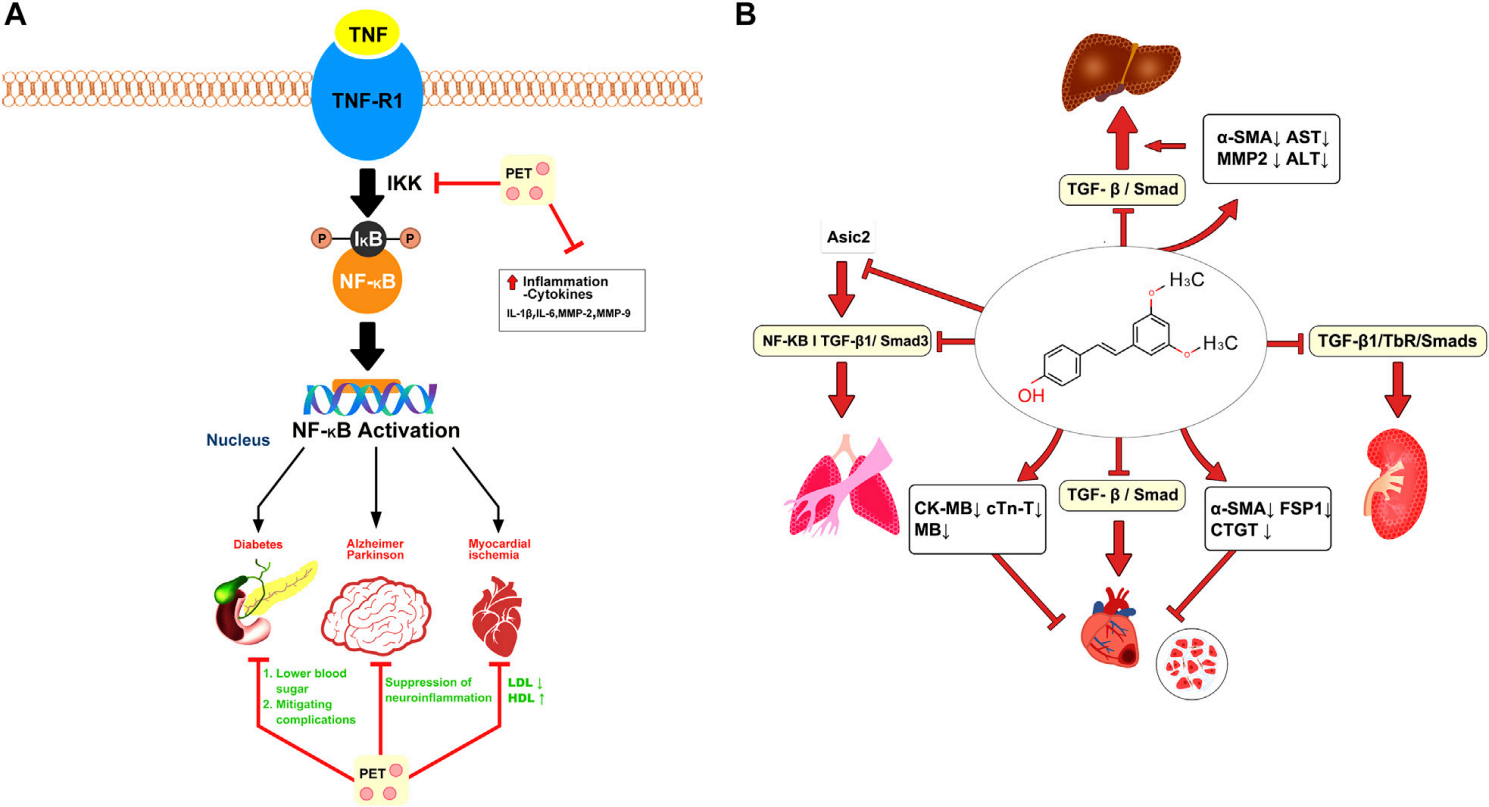
Pterostilbene attenuates inflammatory and fibrotic processes. **(A)** Pterostilbene mitigates inflammation by inhibiting the NF-κB pathway, thereby alleviating conditions such as diabetes, myocardial ischemia, and Parkinson’s disease. **(B)** Pterostilbene alleviates systemic fibrosis (heart, liver, lungs, and kidneys) by inhibiting the TGF/Smad pathway and several other mechanisms.

In animal models of inflammation, PTS has been shown to reduce edema, inflammatory cell infiltration, and cytokine production (Park et al., 2010). Edema, or swelling caused by excess fluid trapped in body tissues, is a common symptom of inflammation (Liu et al., 2019). The anti-edematous characteristics of PTS can assist in reducing swelling and, as a result, lessen the physical discomfort brought on by inflammation (Yan et al., 2021). Another pivotal aspect of inflammation is the infiltration of inflammatory cells into the affected area, leading to tissue damage and furthering the inflammatory response (Mack, 2018). Evidence from animal studies suggests that PTS can attenuate this cell infiltration, thus helping to limit tissue damage and the propagation of the inflammatory response (Liu et al., 2016). The potential anti-inflammatory mechanism of PTS is depicted in Figure 3A.

## Pterostilbene surpasses resveratrol in alleviating inflammation

Compared to RSV, PTS exhibits stronger anti-inflammatory activities. Rats were divided into five groups to investigate the impact of PTS (15 or 30 mg/kg/d) and RSV (30 mg/kg/d) on the progression of non-alcoholic fatty liver disease (NAFLD). One group was fed a standard diet, while the other four were given a high-fat, high-fructose diet supplemented with either PTS or RSV for 8 weeks. The study focused on the effects of these compounds on oxidative stress, inflammation, fibrosis, and pre-carcinogenic stages. The results demonstrated that PTS, particularly at a dose of 30 mg/kg/d, effectively alleviated liver oxidative stress and inflammation caused by the high-fat, high-fructose diet (Gómez-Zorita et al., 2020). Following lipopolysaccharide (LPS) injection, weaned piglets exhibited activated inflammatory responses and severe oxidative stress, as well as enhanced nuclear translocation of NF-κB and increased protein expression of NLRP3 and cleaved caspase. PTS is more effective than RSV in reducing liver damage by targeting the NF-κB/NLRP3 signaling pathway and mitigating inflammation and oxidative stress (Li et al., 2022).

## Anti-inflammatory effect of pterostilbene in diabetes

Inflammation is a crucial factor in the progression of numerous diseases, particularly diabetes, where it serves not only as a hallmark of the disease but also as a driving force for its development and complications (Rohm et al., 2022). In diabetes, sustained high blood glucose levels can induce oxidative stress and inflammatory responses, thereby exacerbating insulin resistance and impacting pancreatic function, further deteriorating the condition (Hurrle and Hsu, 2017). Research has validated the role of PTS in managing diabetic inflammation. Obesity is closely related to diabetes; ómez-Morita found that PTS, at a dosage of 15 mg/kg, was more effective than RSV in reducing weight in rats fed a high-fat diet (Gómez-Zorita et al., 2014). The enhanced performance of PTS is linked to its increased liposolubility, stemming from the substitution of a hydroxyl group with a methoxy group, which boosts its absorption. PTS modulates blood sugar levels, improves insulin response and lipid profiles, and reduces inflammation and oxidative damage in rats with diet-driven obesity and STZ-triggered diabetes by utilizing the PI3K/Akt signaling route (Sun et al., 2019). In addition to effectively controlling high blood sugar, PTS offers potential protective benefits against the complications often associated with diabetes. These complications can range from cardiovascular disease, due to the persistent strain on the heart and blood vessels, to kidney disease, resulting from the body’s struggle to filter blood without sufficient insulin (Millán et al., 2019; Dodda et al., 2020).

## Anti-inflammatory effect of pterostilbene in nervous system disorders

In neurodegenerative diseases, inflammation is a critical factor in disease progression and symptom severity. Neuroinflammation, characterized by the activation of microglial cells and the release of inflammatory cytokines, contributes significantly to neuronal damage and death (Muzio et al., 2021). This inflammatory process is a common pathological feature in conditions such as Alzheimer’s and Parkinson’s disease (King et al., 2019). PTS exhibits neuroprotective effects, as evidenced by its capacity to improve neurological function, lower neurological scoring, and enhance neuronal survival *in vivo*. It has also been shown to boost the number of mature neurons, augment cell vitality, and limit neuronal apoptosis. Pterostilbene’s protective function further extends to the reduction of infarct volume in the brain, the alleviation of cerebral edema, a decrease in the number of activated microglial cells, and the suppression of eNOS and IL-1β expression (Liu et al., 2020). Through attenuating the levels of oxidative stress markers such as 4-hydroxynonenal and 8-hydroxyguanosine, reducing lactate dehydrogenase leakage, reversing elevated MDA concentrations in the ischemic brain hemisphere, and restoring depleted SOD activity, PTS effectively neutralizes oxidative stress, highlighting its influential role as an antioxidant agent (Zhou et al., 2015). PTS has demonstrated neuroprotective effects in preclinical models of Alzheimer’s disease and Parkinson’s disease, potentially due to its anti-inflammatory, antioxidant, and anti-apoptotic properties (Millán et al., 2019; Liu et al., 2020). PTS effectively suppresses neuroinflammation, one of the key pathological features of neurodegenerative diseases, by inhibiting the activation of microglial cells (Zhou et al., 2015). Elevated cholesterol and triglyceride levels heighten the risk of heart-related ailments, such as heart attacks and strokes. Pterostilbene’s potential to enhance lipid markers might serve as a preventive measure against these conditions. Specifically, it's believed to reduce LDL cholesterol, commonly deemed as harmful, while boosting the levels of the beneficial HDL cholesterol (Brenner and Boileau, 2019).

## Antifibrotic effect of pterostilbene

Fibrosis involves an overaccumulation of fibrous connective tissue, stemming mainly from abnormal extracellular matrix buildup. This leads to the creation of scar tissue, potentially causing organ malfunctions and affecting various organs throughout the body (Wynn, 2007). Lee et al. explored the potential of PTS in counteracting inflammation and cell overgrowth effects from dimethylnitrosamine (DMN) in liver fibrosis using male SD rats. Rats were categorized into a control DMN model and two groups receiving different PTS doses. Following a month of DMN injections and PTS treatment, the DMN model group displayed elevated liver enzyme levels and noticeable liver tissue harm. On the other hand, rats treated with 20 mg/kg of PTS showed a decline in these liver enzyme levels and lessened liver damage. This suggests that PTS could potentially enhance liver health, mitigate DMN-triggered liver harm, and decelerate liver fibrosis by targeting hepatic stellate cells (Lee et al., 2013). Gu et al. discovered that PTS and pirfenidone could significantly inhibit the TGF-β1/TbR/Smads signaling pathway in the rat renal cortex. This intervention suppressed fructose-induced epithelial-mesenchymal transition (EMT) in rat proximal tubular epithelial cells, contributing to a reduction in renal tubulointerstitial fibrosis. Moreover, PTS was shown to elevate the expression of pIR, pIRS-1, and pAkt within the rat renal cortex (Gu et al., 2019).

TGF-β1 triggers EMT and ECM buildup, reducing autophagy and cell apoptosis in A549 and AECs cells. Remarkably, PTS at 30 μmol/L lessened the impact of TGF-β1 on pulmonary fibrosis. PTS effectively counteracts EMT and ECM buildup and bolsters cell apoptosis and autophagy in comparison to TGF-β1. Transcriptome sequencing demonstrates a marked decline in ASIC2 protein levels due to PTS. Enhancing ASIC2 expression through plasmid introduction reverses pterostilbene’s effects, accelerating EMT and ECM buildup while suppressing cell apoptosis and autophagy. It is inferred that pterostilbene’s role in alleviating pulmonary fibrosis is linked to ASIC2 downregulation (Peng et al., 2021). KANG et al. explored the effects of PTS on fructose-induced myocardial fibrosis in rats and discovered that it reduced serum markers of cardiac damage, including CK-MB, cTn-T, and M.B. The study implies that PTS may alleviate fructose-induced myocardial injury by inhibiting the TGF-β1/Smad signaling pathway, thereby potentially protecting the heart (Kang et al., 2019). The potential antifibrotic mechanism of PTS is illustrated in Figure 3B.

## Pterostilbene in tumor therapy

Cancer is a complex disease caused by genetic and environmental factors, commonly including genetic mutations, chronic inflammation, lifestyle aspects like smoking and diet, and environmental exposures such as radiation and chemicals (Jin et al., 2019). At its core, the disease often involves genetic mutations, which can be either inherited or acquired over a person’s lifetime. These mutations disrupt the normal cell cycle, leading to uncontrolled cell growth and tumor formation (Nussinov et al., 2022). Chronic inflammation is another key factor, serving as both a cause and a consequence of cancerous growth, creating a vicious cycle that exacerbates the disease. Lifestyle choices play a crucial role as well; habits such as smoking and unhealthy dietary patterns have been strongly linked to increased cancer risk. These lifestyle factors can act as catalysts, accelerating the onset and progression of the disease. Moreover, environmental exposures, notably to radiation and harmful chemicals, can directly damage DNA or create conditions conducive to cancer development. These elements collectively contribute to the complexity of cancer, making it a challenging disease to understand and treat (Jardim et al., 2023). Current treatment modalities primarily consist of surgery, radiotherapy, chemotherapy, and immunotherapy, each with limitations like side effects and drug resistance (Miller et al., 2019). In oncology, traditional chemotherapy agents, while effective, often come with a hefty price tag and a host of adverse effects. PTS emerges as a beacon, both economically viable and associated with reduced side effects. PTS demonstrates superior bioactivity when compared to RSV. Highlighting its potency, PTS, with an impressive IC50 value of 22.4 μmol/L, has shown a remarkably more robust inhibitory impact on the HT-29 colon cancer cell line than RSV, which displays an IC50 value of 43.8 μmol/L (Paul et al., 2009). Such merits have not gone unnoticed, attracting a burgeoning community of researchers eager to unlock its potential further. Empirical studies have highlighted pterostilbene’s impressive anticancer properties prowess, with efficacy demonstrated across a spectrum of malignant neoplasms (Ma et al., 2019). There is growing interest in the prospect of utilizing PTS as a stand-alone therapeutic agent or synergistically alongside existing FDA-approved anti-cancer treatments. Such novel therapeutic strategies underscore a promising horizon for its clinical integration. Delving into its mode of action, it becomes evident that pterostilbene’s antitumor mechanisms are intricate and operate on multiple cellular and molecular fronts. PTS inhibits cancer cell migration and invasion, critical steps in cancer metastasis. PTS may regulate specific enzymes associated with aging and lifespan, which could impact its anticancer properties (Li et al., 2018). This effect has been noted in studies involving breast, lung, and colorectal cancer cells. The tumor-fighting capabilities of PTS can mainly be traced back to several key factors.

## DNA methylation and histone modification

Cancer develops from a complex mix of genetic and environmental influences, involving aberrations in DNA methylation, histone modifications, and the expression of microRNAs (miRNAs) (Saleh et al., 2020). DNA methylation, a key epigenetic mechanism, involves the addition of a methyl group to the DNA molecule, typically at cytosine bases in CpG dinucleotides, which can lead to the silencing of tumor suppressor genes (Haghani et al., 2023). Histone modifications, including acetylation and methylation, change the structure of chromatin, influencing gene expression. PTS and RSV, other dietary phytochemicals with chemopreventive properties, can achieve anticancer effects by altering DNA methylation, modulating histone modifications, or regulating miRNA expression (Lee et al., 2018). Stilbenoid compounds like PTS and RSV can modify the DNA methylation patterns in breast cancer cells through epigenetic mechanisms. This alteration reduces the activity of cancer-boosting NOTCH signaling, limiting the growth and spread of breast cancer cells (Lubecka et al., 2016). When used together, RSV and PTS gradually restore the expression of estrogen receptor-alpha (ERα) in ERα-negative breast cancer cells. This restoration arises from the adjustment of DNA methylation and histone acetylation in these cells, profoundly influencing the functions of DNMT, HDAC, and HAT(Kala and Tollefsbol, 2016). In ovarian carcinoma, PTS potently modulates the phosphorylation of STAT3, subsequently impeding cell cycle advancement and initiating apoptosis, thereby manifesting its pronounced antineoplastic effects (Wen et al., 2018).

## Suppression of cell growth and enhancement of cell apoptosis

PTS impacts several mechanisms linked to cancer advancement. Both *in vitro* and *in vivo* examinations have shown that PTS can restrain tumor cell expansion and trigger apoptosis by influencing different signaling routes, including the PI3K/Akt, MAPK, and NF-κB pathways (Mak et al., 2013; Hsiao et al., 2014; Tong et al., 2021). Perecko et al. found that PTS induced apoptosis in leukemia cells through the MAPK pathway (Hsiao et al., 2014). Increased activity of cyclooxygenase-2 (COX-2) has been noted in lung cancer, and PTE has been identified to regulate the growth and apoptosis of NSCLC cells by focusing on COX-2 (Wang et al., 2023). In conjunction with the HDAC inhibitor vorinostat, PTS proficiently modulates the MTA1/HIF-1α pathway, curbing both cellular proliferation and angiogenesis. This combined action notably hinders the advancement of prostate tumors in murine models, all while showcasing a more favorable toxicity profile. Distinctively, PTS attenuates MTA1 expression within hepatocellular carcinoma cells and concurrently reduces the enzymatic activities of HDAC1 and HDAC2. Such interference destabilizes the intricate MTA1/HDAC molecular assembly, culminating in the heightened acetylation of phosphatase and tensin homolog (PTEN) and the tumor suppressor P53. As a result, a cascade of cellular responses ensues, characterized by inhibited cellular proliferation, amplified apoptotic initiation, cell cycle stagnation, and a marked reduction in cellular migratory and invasive capabilities (Butt et al., 2017; Qian et al., 2017; Qian et al., 2018a). Several studies have shed light on these mechanisms. PTS inhibited the proliferation of prostate cancer cells by modulating the PTEN/Akt pathway (Dhar et al., 2015; Qian et al., 2018b). PTS acts through a unique biochemical process, enhancing the acetylation and reactivation of the tumor suppressor gene PTEN. This effect is achieved by suppressing the MTA1/HDAC complex, which usually deacetylates proteins, thus altering their function. By inhibiting this complex, PTS ensures PTEN remains active, playing a key role in regulating the Akt pathway. The Akt pathway is involved in cell growth and survival, and its overactivity can lead to cancer. Therefore, Pterostilbene’s ability to reactivate PTEN and regulate the Akt pathway highlights its potential as a therapeutic agent in cancer treatment. This mechanism offers a new perspective on targeting cellular pathways for disease therapy, particularly in cancer, where cell growth and survival pathways are often dysregulated.

## Pterostilbene modulates miRNA in tumors

Cancer development is deeply influenced by microRNAs (miRNAs), which are crucial in regulating gene expression post-transcription. They can function as oncogenes, promoting cancer by downregulating tumor suppressors, or as tumor suppressors themselves, inhibiting cancer by targeting oncogenes. This duality in cancer biology makes miRNAs significant for both understanding cancer mechanisms and developing targeted therapies. Their regulation of key cellular processes like cell growth and apoptosis underscores their potential as biomarkers for cancer diagnosis and targets for innovative treatments (Peng and Croce, 2016). PTS exhibits a dose-dependent inhibitory effect on the vitality of endometrial cancer cells, notably inducing apoptosis *in vitro* through the downregulation of miR-663b (Wang et al., 2017). Regarding prostate cancer, the anticancer effects of PTS are manifested through the reduction of miR-17 family members, a phenomenon observed in both experimental and biological settings (Kumar et al., 2017). PTS enhances PTEN expression in liver cancer cells by directly inhibiting miR-19a, which leads to reduced cell growth, cell cycle halt at the S phase, increased apoptosis, and decreased cell invasion (Qian et al., 2018b). Given the importance of miRNAs in cancer diagnosis and outcome prediction, influencing these molecules might be a pivotal feature of pterostilbene’s cancer-fighting capabilities.

## Pterostilbene modulates endoplasmic reticulum stress in tumor

Endoplasmic reticulum stress (ERS) and the subsequent unfolded protein response (UPR) are crucial in the evolution and advancement of cancer. They act as a protective mechanism against physiological stress and can initiate apoptosis if the stress continues (Wang et al., 2012). Consequently, modulation of E.R. stress and manipulation of the UPR present promising methods for novel anticancer therapies. PTS induces the ROS-mitochondria-dependent apoptosis mechanism mediated by ERS, contributing to its anti-cancer activity in human esophageal cancer cells and inhibiting cell proliferation, invasion, and adhesion (Feng et al., 2016). PTS administration can activate ERS and elevate levels of ERS-associated molecules like p-PERK, ATF4, and CHOP. This action facilitates the transfer of Ca^2+^ from the endoplasmic reticulum to the cytoplasm, boosts reactive oxygen species (ROS) signaling, fosters cell apoptosis, and curtails the movement and adhesion of non-small cell lung cancer cells (Ma et al., 2017). Recent studies indicate that PTS enhances the vulnerability of triple-negative breast cancer cells to TRAIL-driven apoptosis by triggering the ROS/ERS signaling route and amplifying DR4 and DR5 expression (Hung et al., 2017). The ability of PTS to modulate ERS could have profound implications for cancer therapy, representing a promising avenue for future research.

## Pterostilbene promotes the autophagy in tumors

Autophagy is a cellular self-digestion process in which a cell recycles portions of its components to maintain homeostasis and adapt to metabolic stress. In terms of cancer, autophagy can play a dual role by promoting cancer cell survival under pressure and preventing tumor progression by maintaining cellular integrity and reducing inflammation and genome instability (Amaravadi et al., 2019). Following exposure to PTS, cell contraction, membrane disruption, and autophagic vesicle genesis are conspicuous in cisplatin-resistant oral cancer cells. Concurrently, an augmentation in the expression of proteins integral to autophagy is observed, thus alluding to the potent autophagy-inducing efficacy of PTS. Importantly, PTS exerts inhibitory control over cell vitality and fusion, a characteristic that is both time and concentration-dependent (Chang et al., 2018). PTS demonstrates its antitumor efficacy through the induction of autophagy, and intriguingly, it does not exert apparent toxic effects on the heart, liver, and kidneys of tumor model mice. This safety profile underscores its potential as a promising antitumor agent (Mei et al., 2018). PTS exhibits its anticancer effects by inducing autophagy, presenting high therapeutic efficacy with minimal side effects. Its effectiveness and safety profile suggest a promising potential for development into an effective anticancer drug and its application in clinical settings.

## Pterostilbene inhibits epithelial-mesenchymal transition and apoptosis in tumors

EMT is a pivotal mechanism facilitating cell migration and invasion, enabling the spread of tumor cells from their origin. PTS interacts with this mechanism, potentially thwarting or dampening EMT (Song et al., 2019). In triple-negative breast cancer cells, PTS hinders their migratory and invasive traits, characterized by a rise in the EMT marker E-cadherin and a decline in Snail, Slug, Vimentin, and ZEB1 (Su et al., 2015). Furthermore, PTS triggers apoptosis in vascular endothelial cells, a crucial strategy to combat cancer spread. Its action is linked to fostering autophagy *via* a surge in intracellular calcium concentration, leading to the activation of AMPKα1 (Zhang et al., 2013). A research piece from 2018 by Chen and others highlighted pterostilbene’s capacity to curtail lung cancer cell metastasis by stimulating autophagy (Chen et al., 2012). In their study, varying PTS dosages reduced tumor size and burden in mice by notable percentages. Pterostilbene’s interaction with the EMT process offers promising avenues in cancer therapies. Specifically, it downregulates NFκB, Twist1, and Vimentin and amplifies E-cadherin expression, markedly reducing tumorigenesis and metastasis in MDA-MB-231 cells when co-cultured with M2 TAMs (Mak et al., 2013).

## Pterostilbene exerts a regulatory role in tumor stem cells

Cancer stem cells (CSCs) form a unique subset within the broader population of cancer cells, characterized primarily by their remarkable ability to self-renew and differentiate into various cell types. These cells are pivotal in the hierarchy of tumor cells due to their distinct characteristics. CSCs are known to be the primary contributors to the resilience of tumors, playing a significant role in drug resistance, recurrence, and metastasis of cancers. Their ability to evade traditional treatments and regenerate the tumor population makes them critical targets in cancer therapy (Zhao et al., 2018). PTS exhibits dose-dependent inhibition of self-renewal capacities and the gene expression of cancer stem cells in lung cancer cells cocultured with M2-TAMs (M2 phenotype tumor-associated macrophages). This effect appears to be mediated through the downregulation of the cancer-promoting gene, MUC1, which suppresses polarization towards M2 and reduces the accumulation of cancer stem cells (Huang et al., 2016). Through an array of signaling pathways, RSV and PTS can target CSCs in various malignancies, including but not limited to breast cancer, colorectal cancer, leukemia, glioblastoma, and lung cancer (Zhang et al., 2018).

## Pterostilbene improves tumor drug resistance

**FIGURE 4:**
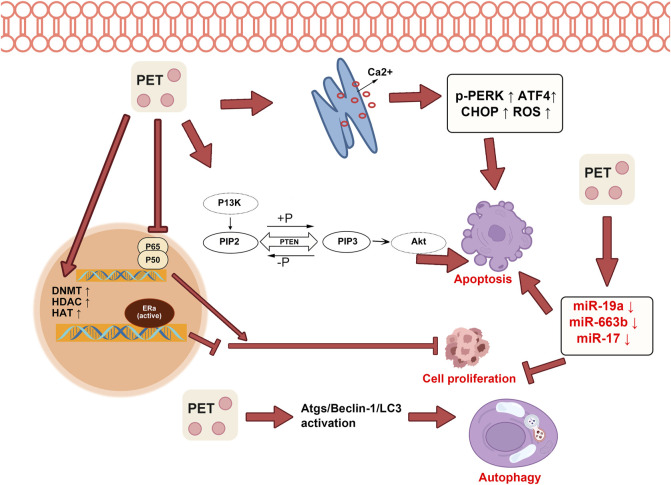
Pterostilbene inhibits tumor cell proliferation and promotes both cell apoptosis and autophagy. Pterostilbene effectively enhances endoplasmic reticulum Ca^2+^ efflux, leading to stimulation of the endoplasmic reticulum stress response and subsequent activation of the PI3K-AKT pathway, which effectively inhibits cell apoptosis. Moreover, it exerts suppressive effects on cell proliferation by inhibiting the NF-κB pathway and modulating the expression of specific microRNAs, including miR-17, miR-19a, and miR-663b. Additionally, pterostilbene promotes tumor cell autophagy by activating the Atgs/Beclin-1/lc3 pathways.

The intricate nature of cancer often gives rise to the emergence of drug resistance, greatly impeding the effectiveness of numerous chemotherapy agents. This resistance can be attributed to diverse mechanisms, such as modifications in drug targets, heightened drug efflux, enhanced DNA repair, and evasion of drug-induced apoptosis (Cree and Charlton, 2017). PTS also reverses multidrug resistance in cancer cells, suggesting its potential role in overcoming chemotherapy resistance (Wang et al., 2023). PTS amplifies cisplatin resistance by elevating LC3-II and Atg12 mRNA levels and boosting Atgs/Beclin-1/lc3-associated signals. This increased autophagy activity hinders the anti-cancer efficacy against human oral cancer CAR cells (Chang et al., 2018). The combined use of PTS and autophagy inhibitors has been shown to improve the therapeutic efficacy of chemotherapy drugs against both chemotherapy-sensitive and chemotherapy-resistant cancer cells. This beneficial effect likely stems from pterostilbene’s capability to trigger autophagy, a cellular recycling process that cancer cells often exploit for survival, especially under stress conditions such as chemotherapy (Hsieh et al., 2014). The possible mechanisms of antitumor pterostilbene are shown in Figure 4.

## Comparative efficacy of resveratrol and pterostilbene in tumor treatment

RSV and PTS, both exhibiting low IC50 values, have been found to downregulate the viral oncogene E6 and tumor protein VEGF levels. In mice treated with pterostilbene, a reduction in tumor size was observed, which was associated with apoptosis. This apoptotic process was indicated by the upregulation of activated caspase-3. On the other hand, resveratrol treatment in mice resulted in cell cycle arrest as evidenced by the downregulation of PCNA. These findings suggest that both resveratrol and pterostilbene have the potential to act as antineoplastic agents in treating HPV E6-positive tumors. They may suppress tumor growth through two distinct mechanisms: pterostilbene inducing apoptosis and resveratrol causing cell cycle arrest (Chatterjee et al., 2019). PTS demonstrated superior efficacy over RSV in suppressing HeLa cell growth, colony survival, and metastasis, and notably inhibiting tumorphere formation and migration in cancer stem-like cells. Its superior inhibitory effect is attributed to enhanced activation through multiple mechanisms, including cell cycle arrest at S and G2/M phases, induction of ROS-mediated caspase-dependent apoptosis, and inhibition of matrix metalloproteinase (MMP)-2/-9 expression (Shin et al., 2020).

## Specific functions of pterostilbene and resveratrol

Obstructive sleep apnea (OSA) is typified by frequent episodes where the upper air passage experiences complete or partial blockage, lasting at least 10 seconds during sleep (Stöwhas et al., 2019). This disorder is widespread among sleep-related health issues. These occurrences trigger a cycle of chronic intermittent hypoxia (CIH), a rise in oxidative stress, and an increase in the levels of cytokines that promote inflammation (Kheirandish-Gozal and Gozal, 2019). The study explored the impact of RSV on lung damage due to CIH, a condition commonly associated with OSA. Following 12 weeks of CIH exposure, rats displayed heightened levels of inflammatory cytokines and increased apoptosis in lung tissues. Treatment with RSV led to reduced inflammation and cell death, as well as enhanced levels of Nrf2 and HO-1 proteins, suggesting that RSV could alleviate lung inflammation and apoptosis related to CIH by triggering the Nrf2/ARE pathway (Lian et al., 2020). Sun et al. demonstrated that resveratrol effectively mitigates myocardial damage associated with CIH by reducing oxidative and endoplasmic reticulum stress and suppressing the NLRP3 inflammasome (Sun et al., 2020).

This study explored the role of PTS, structurally similar to and more active than RSV. In exploring the effects of PTS on brain oxidative stress induced by CIH, a key factor in sleep disorders, the study revealed significant neurological improvements in a CIH mouse model. PTS was found to boost neuronal health, enhance antioxidant levels, and diminish both apoptosis and inflammation within the brain (Liu et al., 2023). Furthermore, it effectively modulated immune responses, increasing anti-inflammatory cells and cytokines, while reducing pro-inflammatory agents. The study highlights Pte’s role in alleviating oxidative stress and correcting immune imbalances in neural cells, achieved through the activation of the p-ERK signaling pathway. Pterostilbene’s effectiveness in tackling the intricate combination of oxidative stress and immune imbalances in OSA showcases its potential as a highly targeted and efficient therapeutic approach. This promising natural compound’s ability to address multiple facets of OSA—from reducing oxidative damage in brain cells to balancing immune responses—signifies a substantial leap forward in developing effective treatments for this widely prevalent sleep disorder. Such multifaceted therapeutic action positions PTS as a noteworthy candidate in the future landscape of OSA management, potentially revolutionizing treatment protocols with its unique and powerful properties.

## Safety and tolerability

The safety of PTS has been extensively studied in preclinical trials. Remarkably, even at a high dose of 3,000 mg/(kg·d), no observable toxic side effects were detected in animal subjects (Ruiz et al., 2009). Furthermore, the current human trials conducted to evaluate the safety of PTS have also yielded positive results. These trials suggest that when administered at therapeutic doses, PTS is safe for human consumption. This is particularly important as it indicates that PTS could be administered to patients without causing severe side effects that could potentially outweigh its benefits (Yang et al., 2017; Sun et al., 2023). However, long-term safety data are lacking, and some studies have raised concerns about potential liver toxicity at high doses (Lacerda et al., 2018). Therefore, further research is needed to determine the optimal dosing and duration of PTS treatment.

## Conclusion

Growing evidence points to pterostilbene’s strong anti-inflammatory and anti-cancer capabilities, highlighting its potential as a treatment for inflammatory and cancer-related conditions. Its antioxidant and neuroprotective attributes further amplify its therapeutic promise. While preliminary clinical studies are encouraging, comprehensive and rigorous trials are imperative to thoroughly assess its therapeutic benefits and safety. As studies progress, delineating the mechanisms of pterostilbene’s actions and determining the best dosage and delivery techniques for various medical uses will be essential.
